# Sonication–Freezing–Assisted Extraction of Chia Seed Mucilage: Functional and Structure–Rheology Relationships and Molecular Weight Determination

**DOI:** 10.3390/gels12050394

**Published:** 2026-05-02

**Authors:** Monserrat Sanpedro-Díaz, Esteban F. Medina-Bañuelos, Ma. de la Paz Salgado-Cruz, Benjamín M. Marín-Santibáñez, Alitzel Belem García-Hernández, Ana Luisa Gómez-Gómez, Diana Maylet Hernández-Martínez

**Affiliations:** 1 Departamento de Ingeniería Bioquímica, Escuela Nacional de Ciencias Biológicas, Instituto Politécnico Nacional, U. P. Adolfo López Mateos, Ciudad de México 07738, Mexico; msanpedrod1700@alumno.ipn.mx (M.S.-D.);; 2 Área de Química, Departamento de Ciencias Básicas, Universidad Autónoma Metropolitana Azcapotzalco, Ciudad de México 02128, Mexico; 3 Escuela Superior de Ingeniería Química e Industrias Extractivas, Instituto Politécnico Nacional, U. P. Adolfo López Mateos, Ciudad de México 07738, Mexico; 4 SECIHTI-Centro de Investigación en Química Aplicada, Parque de Investigación e Innovación Tecnológica, Nuevo León 66628, Mexico; ali_ialee@outlook.com; 5 Departamento de Biofísica, Escuela Nacional de Ciencias Biológicas, Instituto Politécnico Nacional, U. P. Santo Tomás, Ciudad de México 11340, Mexico; dhernandezmar@ipn.mx

**Keywords:** chia seed mucilage, hydrogel, pseudoplastic, viscoelastic, molecular weight, rheological properties

## Abstract

Chia seed mucilage (CSM) is a promising plant-derived hydrocolloid characterized by unique physicochemical and functional properties that are strongly influenced by the extraction methodology. In this research, an optimized sonication–freezing-assisted extraction (SFAE) process was developed to obtain mucilage while preserving its structural integrity. Results indicate that the extracted mucilage has a high total dietary fiber content of 75.87% and a moderate protein level of 8.71%. Fourier transform infrared spectroscopy (FTIR) confirmed the presence of hydroxyl and ionized carboxylate (COO^−^) groups associated with uronic acids, highlighting the anionic and polyelectrolyte nature of the system. Rheological characterization of optimized-CSM revealed Newtonian behavior in dilute solutions, indicating minimal intermolecular interactions and permitting accurate measurement of intrinsic viscosity and viscosity-average molecular weight. A critical overlap concentration (c** ≈ 0.2% *w*/*v*) was identified, marking the transition to semi-dilute regimes, chain entanglement, and the onset of shear-thinning and viscoplastic behavior. Functionally, the optimized-CSM exhibited high water holding capacity and competitive emulsifying properties (emulsion activity index (EAI): 62.50%; emulsion stability index (ESI): 49.32%), attributed to synergistic interactions between proteins and polysaccharides. Overall, this work provides new insights into how processing conditions influence the chemical composition and molecular structure, which fundamentally govern the rheological and functional performance of CSM. These findings underscore its potential as a versatile hydrocolloid for food and biomedical applications.

## 1. Introduction

In recent years, plant-derived polysaccharides have emerged as key components in the design of advanced hydrogel-based systems. These biopolymers can be assembled into three-dimensional macromolecular networks that absorb large volumes of solvent, making them highly relevant for applications in biomedicine, food structuring, and the development of soft materials [1,2,3,4]. The macroscopic performance of such hydrogels, particularly their viscoelasticity and water-binding capacity, is fundamentally governed by the molecular architecture of the constituent chains. Parameters such as molecular weight (*M_w_*), polydispersity index (PDI), and chain arrangement determine the degree of packing and connectivity within the polymer network [5,6,7].

Among emerging biopolymers, chia seed mucilage (CSM; *Salvia hispanica* L. chia) has gained importance as a polysaccharide capable of forming hydrogels. Upon hydration, chia seeds release a complex heteropolysaccharide of high molecular mass, structurally characterized by a branched topology with a tetrasaccharide repeating unit. This unit comprises a backbone of (1à4)-ϐ-D-xylopyranosyl residues and side chains of 4-O-methyl-D-glucuronic acid, which promote extensive interchain entanglement and hydrogen bonding. These interactions enable the formation of physically cross-linked networks with tunable rheological properties [1,8,9,10].

However, the functionality of CSM-based hydrogels is highly dependent on the primary polymer structure, which can be compromised during extraction. The molecular weight of CSM is a decisive factor in chain entanglement, water retention capacity, viscoelastic behavior, and gel strength. As summarized in Table 1, reported molecular weight, *M_w_*, values for CSM vary significantly, ranging from 4.9 × 10^5^ to 2.34 × 10^6^ g/mol [11,12]. This variability reflects the strong influence of extraction and purification methodologies, since processing conditions can impose thermal, mechanical, and chemical stresses that induce depolymerization, conformational rearrangements, or fractionation of mucilage components. Although intensification strategies such as sonication [9,13,14], microwave-assisted extraction [8,15], and high-speed shear treatments have been proposed to improve mass transfer and yield [16]. These methods often involve high energy inputs that may adversely affect polymer integrity. For instance, high-intensity ultrasonication, especially when applied through probe-type systems, as reported by Urbizo-Reyes [9], generates localized shear gradients and transient hot spots that promote chain fragmentation in polysaccharides. In this sense, the degree of degradation strongly depends on the sonication mode. A probe-type sonicator (direct, high-power) delivers concentrated energy directly into the sample, whereas ultrasonic baths (indirect, lower-power) provide a more homogeneous but lower-intensity energy distribution. Despite these differences, even moderate cavitation can reduce hydrodynamic volume and hinder the formation of cohesive, percolating networks [6,11]. Therefore, while sonication can improve extraction efficiency, its application must be carefully optimized to avoid compromising the structural attributes that govern hydrogel functionality.

Moreover, variability in reported *M_w_* values reflects differences in measurement techniques. Methods such as SEC-MALLS-Viscosimetry provide a direct determination of the radius of gyration, whereas relative calibrations using polyethylene glycol/polyethylene oxide (PEG/PEO) yield less accurate estimates. Viscosimetry approaches remain widely used to estimate the viscosity-average molecular weight, *M_v_* [11]. Although not absolute, the determination of intrinsic viscosity [η] via the Huggins and Kraemer equations remains a rapid and reproducible alternative. For high-molecular-weight polymers, however, the Newtonian regime is narrow, and shear-induced errors can compromise accuracy. To address this, Marín-Santibáñez et al. [7] proposed using a double-gap Couette flow geometry, which, when coupled with an appropriate viscosimeter/rheometer, can detect viscosity changes as small as 0.025 mPa·s. This accurate method helps define the limits of the dilute regime and ensures that *M_v_* is calculated solely from Newtonian data, without empirical corrections.

Although the potential of chia seed mucilage as a functional hydrocolloid is well recognized, a critical challenge remains: achieving high extraction yield while preserving polymer structural integrity, which ultimately determines its functional performance. In this context, the present study introduces a sonication–freezing-assisted extraction (SFAE) method designed to balance process efficiency with molecular preservation. The approach integrates lower sonication to minimize chain scission, freezing-assisted structuring to promote gentle detachment, and conventional oven drying as a cost-effective alternative to lyophilization. In this context, the challenge does not lie in the use of sonication itself, but rather in precise control of the energy input and its integration within a broader extraction strategy. Consequently, alternative approaches are required to minimize dependence on high-intensity mechanical disruption while still promoting efficient polymer release. Among these, freeze-assisted processes represent a particularly promising opportunity, as the formation of ice crystals induces structural weakening and facilitates the detachment of polysaccharides without severe shear stresses.

Therefore, this work establishes a comprehensive structure–function framework that links chemical composition, intrinsic viscosity, and viscosity-average molecular weight to rheological behavior across different concentration regimes. The findings provide deeper insight into how controlled processing modulates molecular conformation, governs intermolecular interactions, and shapes the technological functionality of chia mucilage. Collectively, this study highlights the importance of tailoring extraction strategies to preserve polymer architecture, thereby unlocking the full potential of CSM as a versatile hydrocolloid for advanced applications in food, biomaterials, and hydrogel design.

## 2. Results and Discussion

### 2.1. Optimization and Yield

**Figure 1:**
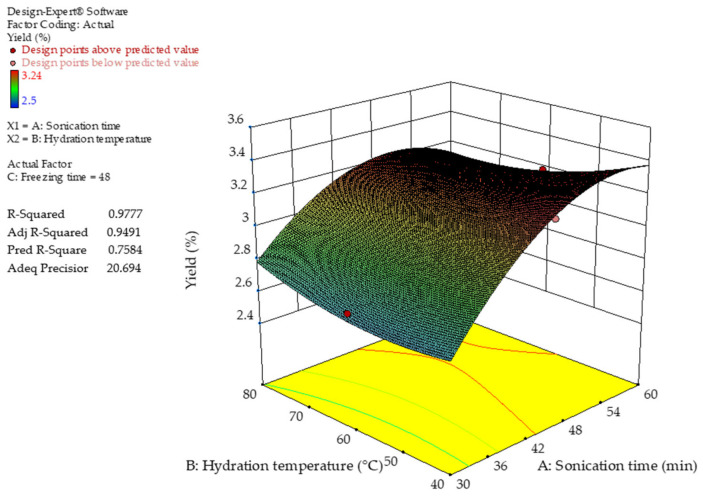
Three-dimensional response surface plot of the extraction yield of chia seed mucilage (CSM). Factors A—sonication time and B—hydration temperature.

**Figure 2:**
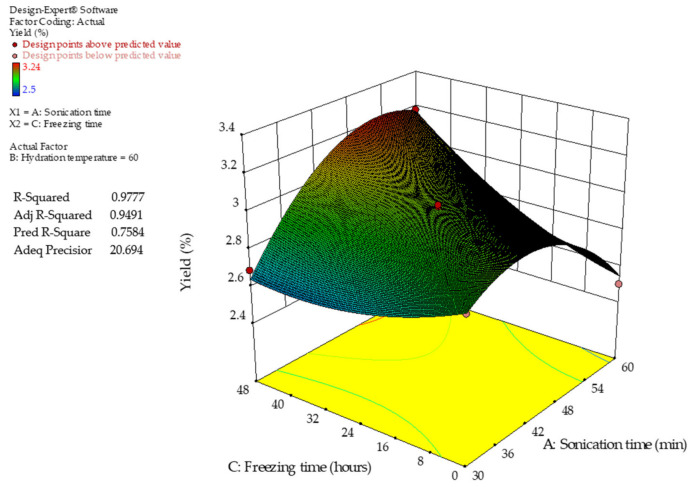
Three-dimensional response surface plot of the extraction yield of chia seed mucilage (CSM). Factors: A—sonication time and C—freezing time.

Figure 1 and Figure 2 show the response surface plots illustrating the combined effects of sonication (Factor A), hydration temperature (Factor B), and freezing time (Factor C) on the extraction yield of chia seed mucilage. The results exhibited nonlinear behavior, characteristic of a quadratic model, with both individual and interaction effects among the evaluated factors. The final equation in terms of coded factors is given in Equation (1).

(1) $$\mathrm{Yield}\;\left(\%\right)=2.96+0.056\;A-0.015\;B+0.10\;C-0.093\;AB+0.21\;AC-0.012\;BC-0.25\;A^{2}+0.081\;B^{2}+0.089\;C^{2}$$

The positive linear coefficients for sonication (A) and freezing time (C) indicate that increasing these variables enhances extraction yield, whereas hydration temperature (B) shows a minor negative linear effect. Among the interaction terms, the positive AC coefficient suggests a strong synergistic effect between sonication and freezing, while the negative AB interaction indicates that high sonication combined with high temperature may reduce yield. Additionally, the significant negative quadratic term of A^2^ confirms the existence of an optimal sonication level, beyond which yield decreases, likely due to polysaccharide degradation.

The robustness of the model was supported by statistical parameters. The coefficient of determination (R^2^ = 0.9777) indicates that the model explains most of the variability in the experimental data. The difference between the adjusted R^2^ and predicted R^2^ was small (ΔR^2^ = 0.19), remaining below the commonly accepted threshold of 0.20, indicating good agreement between the explanatory and predictive capabilities of the model. Therefore, the model can be considered reliable for prediction within the experimental domain. Furthermore, the Adeq Precision value (20.694) indicated a strong signal-to-noise ratio, supporting the suitability of the model for navigating the design space and identifying optimal extraction conditions.

Figure 1 shows that increasing sonication time from low levels (~30 min) to intermediate values (40–50 min) improved extraction yield. This behavior is attributed to acoustic cavitation, which disrupts cellular structures and facilitates polysaccharide release. However, longer sonication times resulted in a slight decrease in yield, likely due to fragmentation of the polysaccharide chains into lower-molecular-weight fractions. These smaller molecules remain soluble in the aqueous-alcohol medium and may not precipitate efficiently during recovery, thereby reducing the final yield.

Regarding the combined effect of hydration temperature and sonication (Figure 1), intermediate temperatures (60–70 °C) enhanced yield, consistent with previous studies reporting that mucilage hydration promotes supramolecular organization, such as entangled nanofibers and coiled conformations, which facilitate mucilage diffusion into the medium [17,18]. Conversely, higher temperatures (~80 °C) decreased the yield, likely due to thermal degradation of polysaccharide chains and disruption of weak intermolecular interactions, such as hydrogen bonds. Elevated temperatures also increase vapor pressure, altering cavitation dynamics and reducing bubble collapse intensity, thereby decreasing extraction efficiency. The combined effect of high temperature and prolonged sonication further accelerated mucilage degradation [19].

Seed hydration is a dynamic and competitive process. In early stages, mucilage release predominates; however, at higher temperatures, increased water absorption by the seed matrix may favor tissue hydration over mucilage diffusion, thereby limiting extraction efficiency.

Figure 2 illustrates the combined effect of sonication time (factor A) and freezing time (factor C) on extraction yield. Sonication had a strong positive effect, with yield increasing from 30 to 60 min and reaching a range of 3.1–3.2%. This behavior is consistent with enhanced acoustic cavitation, which promotes cell wall rupture and mass transfer. In contrast, freezing time showed a non-linear effect: moderate freezing improved yield because ice crystal formation disrupted the seed matrix, whereas prolonged freezing reduced yield, particularly at lower sonication levels, likely due to matrix compaction and reduced mucilage diffusivity.

The interaction between sonication and freezing revealed that the negative effects of prolonged freezing were partially mitigated at higher sonication times, suggesting that increased mechanical energy enhances mass transfer and compensates for diffusional limitations. Overall, the highest extraction yields were achieved under high sonication and freezing conditions, indicating a synergistic interaction between these variables.

Collectively, response surface analysis indicates that extraction yield is governed by the combined effects of sonication, hydration temperature, and freezing time. Intermediate processing conditions enhance mucilage extraction efficiency. However, although ultrasound improves yield, it may also reduce the molecular weight of polysaccharides. High-molecular-weight chains are more susceptible to ultrasonic breakage, which can influence the techno-functional properties of the extracted mucilage. Therefore, correlating molecular structure with functional properties, particularly gel-forming capacity, remains essential.

### 2.2. Extraction of Chia Seed Mucilage Optimization

**Figure 3:**
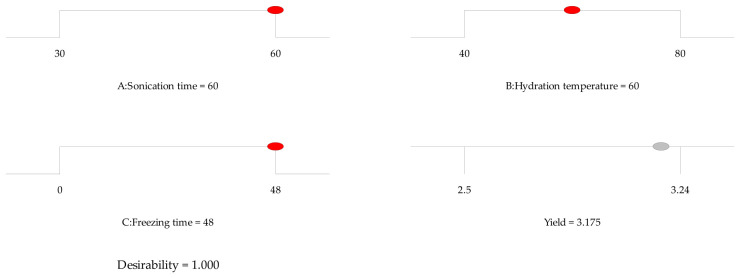
Optimal conditions for maximum chia seed mucilage (CSM) extraction yield through Box–Behnken surface response methodology using Design Expert V. 10.

The optimal extraction conditions were determined by maximizing yield through a numerical optimization approach implemented in Design-Expert^®^. This procedure identified an optimal region that achieves the desired response (Figure 3).

Under these conditions, the optimal parameters were a sonication time of 60 min, a hydration temperature of 60 °C, and a freezing time of 48 h, yielding an overall desirability of 1.00. To assess the predictive capability of the model, the optimized conditions were experimentally validated.

The extraction yield of optimized-CSM was 3.39 ± 0.24%, showing good agreement with the model predictions. This value is also comparable to the 3.8% yield reported by Timilsena et al. [12]. However, a direct comparison should be made with caution due to differences in extraction protocols. Timilsena et al. [12] employed extensive multi-step purification, including Soxhlet defatting, repeated ethanol washings, and dialysis, to obtain high-purity mucilage for molecular-weight characterization rather than for process scalability.

In contrast, lower yields have been reported by Wang [13] and Goh et al. [11] (2.11% and 1.2%, respectively), likely due to variations in extraction and purification strategies, and the use of freeze-drying, which may increase material losses and operational costs. Conversely, Li et al. [16] reported a higher yield (6.49%) attributed to the application of high-speed shearing, which can mechanically disrupt the seed coat and may promote the co-extraction of intracellular components, such as lipids, ash, and proteins, potentially compromising the purity of the extracted mucilage.

Overall, the results demonstrate that the SFAE method provides a balanced approach between extraction efficiency and process simplicity. While some methodologies have been reported to achieve higher yields or purity, they often rely on intensive mechanical treatments or complex purification steps that may limit scalability and increase processing costs. In contrast, the optimized conditions identified in this study enable efficient recovery of mucilage while preserving its structural integrity to a reasonable extent. This highlights the importance not only of maximizing yield but also of considering the relationships among extraction conditions, molecular structure, and techno-functional properties.

### 2.3. Physicochemical Characterization of Optimized-CSM

The macroscopic appearance of the optimized-CSM was evaluated through visual inspection and instrumental color measurements. These parameters were correlated with mucilage purity and chemical composition, as determined by proximate analysis and Fourier transform infrared (FTIR) spectroscopy. This characterization provides an insight into how the optimized extraction conditions influence the structural and compositional attributes of CSM, which are linked to its techno-functional properties and potential applications.

#### Appearance and Color Measurements

**Figure 4:**
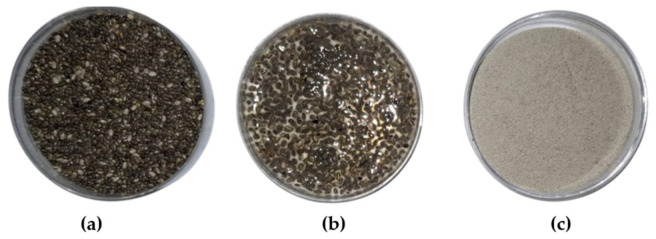
Macroscopic appearance of chia seed mucilage during the extraction process: (**a**) dry seeds, (**b**) hydrated seeds showing mucilage formation, and (**c**) dried mucilage after extraction.

One important parameter is the macroscopic appearance of optimized-CSM. As shown in Figure 4c, the material was obtained as a fine, homogeneous gray powder. Chia seeds typically appear darker when dried (Figure 4a); however, they become lighter and more translucent upon hydration. This change can be attributed to changes in refraction within the hydrated mucilage layer, which reduce light scattering and enhance light transmission.

Instrumental color analysis in the CIEL*a*b* color space revealed a lightness (L*) of 64.61 ± 0.49, with chromatic coordinates a* = 1.44 ± 0.08 and b* = 8.23 ± 0.18. These results indicate a relatively light-colored material, in contrast to the darker L* values (21.23 to 41.22) reported by Wang et al. [13] for mucilage obtained via heat- and ultrasound-assisted extraction. This darker coloration has been associated with the diffusion of natural pigments and tannins from the seed coat (tegument) into the mucilage matrix during prolonged or high-temperature extraction processes [12,20]. In this context, the higher L* value observed for optimized-CSM suggests that the extraction conditions effectively limited pigment diffusion. Furthermore, the yellowness (b*) value of 8.23 is comparable to that reported by Timilsena et al. [12] for a highly purified mucilage obtained by dialysis, indicating that the proposed method achieves similar color quality without extensive purification.

The macroscopic appearance and color of optimized-CSM are closely related to its chemical composition and purity. The high lightness and low chromatic coordinates indicate minimal co-extraction of pigments, phenolic compounds, and other seed coat constituents, consistent with a predominantly polysaccharide-based mucilage. This interpretation is further supported by proximate composition.

### 2.4. Proximate Composition of Chia Mucilage

The moisture content of CSM powder (12.23 ± 0.16%, Table 1) was higher than values reported in other studies, likely due to oven drying rather than lyophilization. This can be explained by the fact that freeze-drying is more effective at removing both free and bound water, whereas conventional drying may be limited by the formation of a dense polysaccharide matrix that restricts water diffusion and evaporation [21]. In polysaccharide-rich systems such as mucilage, compact or semi-glassy structures can significantly reduce water mobility, thereby hindering water removal and leading to higher residual moisture content. Moreover, this behavior is also intrinsically related to the strong water-binding capacity of chia mucilage, which, due to its hydrophilic nature and abundant hydroxyl groups, allows the polysaccharide network to retain significant amounts of bound water, even after drying [22,23].

Total dietary fiber (TDF) was the major component of the extracted CSM (75.87 ± 0.76%), followed by ash, protein, lipids, and carbohydrates. As shown in Table 2, the chemical composition reported by other authors is presented for comparison; however, many studies do not explicitly quantify dietary fiber, it is incorporated into the carbohydrate fraction instead. The high TDF content observed here reflects the intrinsic nature of chia mucilage, which is primarily composed of heteropolysaccharides. As previously reported [11,20,24], these polysaccharides consist mainly of arabinose, xylose, glucose, and uronic acids, forming a complex structural network that is quantified as dietary fiber. Therefore, the predominance of TDF confirms the polysaccharide-rich composition of the mucilage and supports its classification as a hydrocolloid with high functional potential.

The lack of specific quantification of dietary fiber in other studies may lead to overestimation of carbohydrate content (Table 2), limiting the accurate interpretation of mucilage composition. In contrast, the methodology used in this study enables precise characterization of the structural polysaccharide fraction and, consequently, a more reliable evaluation of techno-functional properties.

The extracted mucilage exhibited protein and ash contents of 8.71% and 9.08%, respectively. These values are higher than those reported by Wang et al. [13] for purified mucilage, which was subjected to protease-assisted enzymatic hydrolysis to remove protein fractions. However, Wang et al. [13] also reported protein and ash contents in the ranges of 5.12–12.05% and 8.73–11.32%, respectively, for unpurified mucilage obtained by sonication and thermal treatment, consistent with the values observed in this study.

In contrast, the protein content reported here is significantly lower than the 25.20% observed by Silva et al. [14] for samples subjected to high-intensity ultrasound (30 min/U30). This discrepancy can be attributed to the combined effects of sonication intensity and extraction temperature on the seed matrix and protein behavior. High-intensity probe sonication generates strong cavitation and shear forces that disrupt the seed coat, promoting the release of intracellular compounds, including storage proteins and minerals, into the mucilage fraction [14]. By comparison, the sonication conditions used in the SFAE method appear less severe, favoring the selective release of mucilage from the seed epidermis while limiting excessive cellular disruption. Additionally, the use of a moderate temperature (60 °C) in combination with sonication may have contributed to partial denaturation of the protein. The synergistic effect of thermal treatment and acoustic cavitation can induce conformational changes, protein aggregation, and protein unfolding, reducing their solubility and limiting incorporation into the extracted mucilage. Consequently, the protein content is affected by the processing conditions.

Overall, compositional differences reported in previous studies suggest that extraction conditions strongly influence the balance between yield and purity of chia seed mucilage (Table 2). More intensive extraction methods, such as high-power sonication or prolonged thermal treatments, tend to increase the release of intracellular components, including proteins, lipids, and minerals, resulting in higher apparent yields but reduced compositional purity. In contrast, the SFAE method appears to promote a more selective recovery of the polysaccharide-rich fraction, as reflected by the high total dietary fiber content and the moderate levels of protein and ash. These findings support the commonly reported trade-off between extraction efficiency and purity in hydrocolloid systems.

### 2.5. Fourier Transform Infrared Spectroscopy (FTIR) Analysis

To further elucidate the relationship between extraction conditions and the structural features of the optimized-CSM, Fourier transform infrared (FTIR) spectroscopy was performed. This technique provides insights into the functional groups present in the biopolymer matrix and allows the assessment of its structural integrity after the SFAE process. FTIR also enables the identification of molecular interactions among polysaccharides, proteins, and residual components, which are directly linked to the techno-functional properties of mucilage.

**Figure 5:**
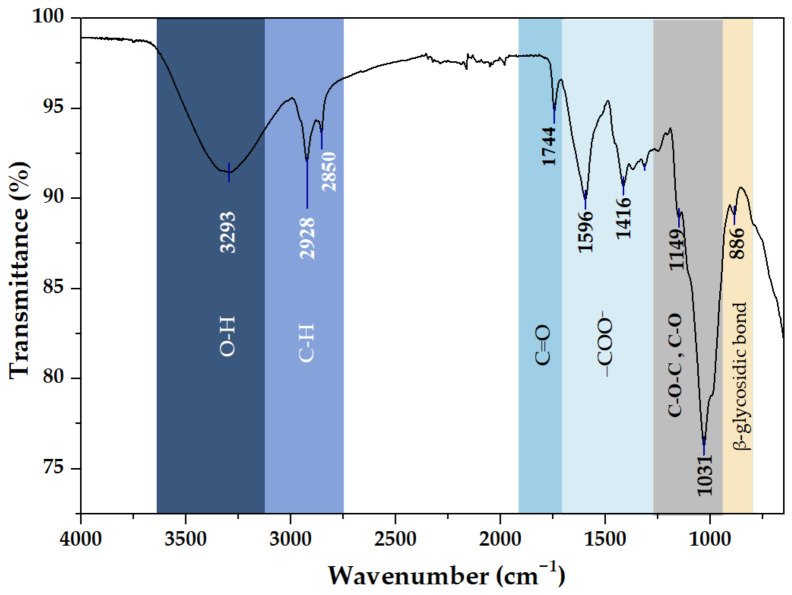
FTIR spectra of optimized chia seed mucilage extraction through sonication–freezing-assisted method.

The FTIR spectrum of optimized-CSM (Figure 5) exhibited several characteristic absorption bands between 608 and 3293 cm^−1^. The prominent band near 3300 cm^−1^ corresponds to O-H stretching vibrations associated with hydroxyl groups in the carbohydrate structure. This feature reflects extensive hydrogen-bonding interactions and may include contributions from residual moisture [25,26,27]. Its high intensity indicates that the SFAE process effectively preserved the hydrophilic sites. The absorption bands at 2928 and 2850 cm^−1^ are attributed to asymmetric and symmetric C-H stretching vibrations of aliphatic groups present in the polysaccharide backbone. A distinct band at 1744 cm^−1^ is associated with C=O stretching vibrations, which may arise from esterified groups or residual lipids. Additionally, the strong bands at 1596 and 1416 cm^−1^ correspond to asymmetric and symmetric stretching of the carboxylate group (COO^−^), confirming the presence of uronic acid residues within the mucilage structure. These groups are characteristic of anionic polysaccharides [25,28]. Carboxylate groups originate from deprotonated carboxylic acids and are stabilized by resonance, enhancing polarity and water affinity.

Although these functional groups are intrinsic to the polysaccharide structure, sonication may further expose them by disrupting cell walls and releasing uronic acid-rich fractions, thereby enhancing hydration, intermolecular interactions, and functional properties, including water-binding capacity and gel formation.

Notably, the broad feature centered at 1596 cm^−1^ may include contributions from the Amide I and Amide II bands, suggesting the presence of protein fractions that interact with the polysaccharide matrix. This spectral overlap supports compositional data and indicates the integration of proteins within the hydrophilic matrix, which may contribute to the emulsifying properties of optimized-CSM.

Finally, in the fingerprint region, the strong band at 1031 cm^−1^ corresponds to C-O-C stretching vibrations, indicating abundant structural alcohols, while the signal at ~886 cm^−1^ is associated with β-glycosidic linkages, confirming the preservation of the polysaccharide backbone in the extracted mucilage [26].

### 2.6. Intrinsic Viscosity and Viscosity-Average Molecular Weight

The rheological behavior of CSM solutions is closely related to their chemical composition and molecular structure. The high total dietary fiber content, together with the presence of ionized carboxylate groups (COO^−^) identified by FTIR, contributes to the polyelectrolyte nature of the mucilage.

**Figure 6:**
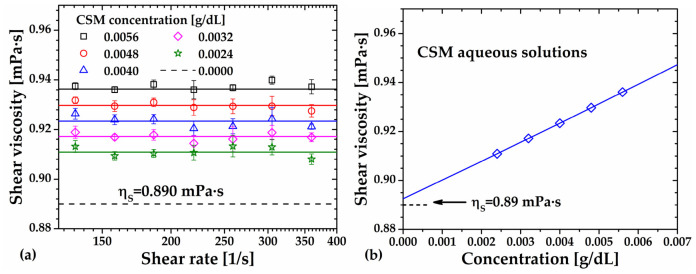
(**a**) Viscosity curves of chia seed mucilage (CSM) solutions at different concentrations. The solid lines represent the Newton viscosity equation fitted to experimental data. The dashed line represents the viscosity of water (NIST). (**b**) Viscosity of CSM solutions as a function of concentration.

Viscosity curves of aqueous CSM solutions in the concentration range of 0.002 to 0.006 g/dL are shown in Figure 6, obtained by varying the shear rate from 125 to 425 s^−1^. The dashed line represents the viscosity of water (solvent) at 25 °C (NIST database) [29]. Error bars indicate the standard deviation of four replicates. As illustrated in Figure 6a, the viscosity of all CSM solutions was independent of shear rate, confirming the Newtonian flow behavior and suggesting the absence of polymer–polymer interactions. This observation is critical for the reliable determination of viscosity-average molecular weight (*M_v_*). All flow curves were fitted to the Newton viscosity equation with correlation coefficients R^2^ ≥ 0.9999, and the solid lines in Figure 6a represent the viscosity of each CSM solution.

Additionally, Figure 6b shows that the viscosity of CSM solutions decreases according to a second-degree polynomial trend and approaches that of the solvent, with an error of only 0.25%, as the polymer concentration tends to zero. This behavior is in agreement with previous reports for polymer and polyelectrolyte solutions [7,30].

**Figure 7:**
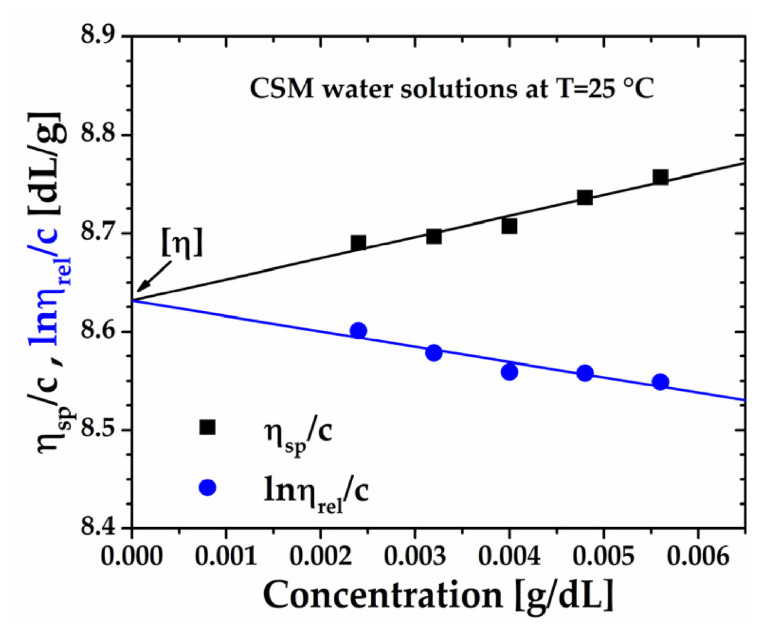
Huggins (squares) and Kraemer (circles) lines of purified chia seed mucilage (CSM) aqueous solutions at 25 °C. The solid lines represent the corresponding fittings.

Using the viscosity data, the reduced viscosity (*η_sp_*/*c*) and inherent viscosity (${ln(\eta }_{rel})/c$) were calculated using Equations (A1)–(A4) (Appendix A) and plotted against concentration in Figure 7. Both relationships were well described by the Huggins and Kraemer models, with correlation coefficients R^2^ > 0.940 and 0.89, respectively. Extrapolation to infinite dilution yielded an intrinsic viscosity $\left[\eta \right]=8.63$ dL/g. Additionally, the sum of the Huggins and Kraemer constants ($k^{\prime }+k^{\prime \prime }=0.49$) was consistent with theoretical expectations.

Using the Mark–Houwink–Sakurada equation, $\left[\eta \right]=0.000152\cdot M_{v}^{0.803}$, reported by Timilsena et al. [12], the viscosity-average molecular weight of CSM was calculated to be 832.7 kg/mol. This value is comparable to those reported for depolymerized chia seed polysaccharides [12]. Minor differences in the viscosity-average molecular weight relative to the literature can be attributed to variations in ultrasound conditions, particularly processing time and intensity, which induce mechanical and cavitation effects that promote chain scission and partial depolymerization of the polysaccharide backbone, thereby affecting the molecular weight distribution [31]. Additionally, variability may arise from differences in purification protocols, seed origin, and degree of maturation, all of which influence the structural and compositional characteristics of plant-derived polysaccharides. Therefore, the combined effect of intrinsic chemical composition and controlled extraction conditions governs the molecular conformation in solution, ultimately determining the hydrodynamic behavior and functional performance of the mucilage [32].

### 2.7. Weight-Average Molecular Weight

**Figure 8:**
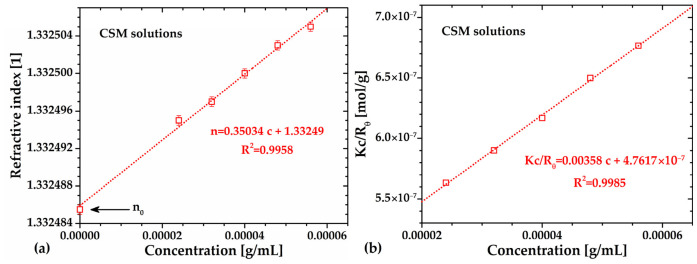
(**a**) Refractive index as a function of *c* for *dn/dc* determination. The error bars represent the difference between the minimum and maximum values recorded over 1 min of measurement for two different samples at a constant temperature. (**b**) Debye light-scattering plot for determination of the weight-average molecular weight.

The weight-average molecular weight (*M_w_*) of CSM was determined by static light scattering (SLS) after viscosity measurements, ensuring that *M_w_* was calculated from purely Newtonian solutions (Figure 6a). Prior to SLS analysis, the refractive index, *n*, of all the Newtonian CSM solutions was measured using a high-resolution refractometer (1 × 10^−6^; Section 4.4.2). The resulting plot (Figure 8a) showed a linear trend that extrapolates to the refractive index of water, $n_{0}=1.332485$, with a slope of *dn/dc*=0.35034 mL/g. This value was used to calculate *Kc/R_θ_* and construct the Debye plot for $M_{w}$ determination (see Section 4.4.2).

The Rayleigh ratio (*R_θ_*) was obtained using the refractive indices of water and toluene ($n_{T}=1.494009$), as reference fluids, with $R_{\theta T}=1.14574\;{\times \;10}^{-5}\;{cm}^{-1}$. As shown in Figure 8b, the relationship between the amount *Kc/R_θ_* and CSM concentration was well described by the Debye linear equation with R^2^ = 0.9997, yielding a second virial coefficient $A_{2}=0.00179$ mol·mL/g^2^ and $M_{w}=2.1\;\times \;1$0^6^ g/mol. The positive *A*_2_ value indicates favorable polymer–solvent interactions, confirming that water is a good solvent for CSM.

SLS measurements also enabled the estimation of the critical overlap concentration, c*, which marks the transition from Newtonian to shear-thinning or viscoelastic behavior due to polymer–coil interactions. Here, the value of $c^{*}$ was calculated as $c^{*}\sim \frac{1}{A_{2}\cdot M_{w}}\sim 0.00027$ g/mL or $c^{*}\sim 0.027$ g/dL, consistent with viscosity data concentrations of *c* = 0.0056 and 0.050 g/dL (see Figure 6a and the flow curves shown in Section 2.9, respectively). These results demonstrate strong agreement between viscosity and SLS measurements, confirming the reliability of both *M_v_* and *M_w_* determinations.

The *M_w_* obtained for CSM is comparable to that of other polysaccharides, such as xanthan gum ($M_{w}=4.650\;\times \;10^{6}$ g/mol) and guar gum ($M_{w}=1.300\;\times \;10^{7}$ g/mol) [33,34], but significantly higher than that of crude chia seed gum ($M_{w}=3.985\;\times \;10^{5}$ g/mol) extracted at water boiling temperatures [16]. Elevated extraction temperatures accelerate glycosidic bond hydrolysis, leading to rapid depolymerization of polysaccharides. Thus, the moderate temperature (60 °C) and low sonication power used during the SFAE process preserved polymer integrity and resulted in higher *M_w_* values.

It is well known that the functional properties of the CSM depend on its molecular weight, which is a decisive factor in chain entanglement, water retention capacity, viscoelastic behavior, and gel strength. The revised works in Table 1 show that the molecular weight, *M_w_*, of CSM obtained from various extraction and purification methods, may vary from 4.9 × 10^5^ to 2.34 × 10^6^ g/mol [11,12]. For instance, Urbizo-Reyes [9] reported that high-temperature and high-intensity ultrasonication, especially when applied via probe-type systems, generate localized shear gradients and transient hot spots that promote chain fragmentation and depolymerization. Therefore, by comparing the *M_w_* of the optimized-CSM via the SFAE process to reported *M_w_* values, it can be inferred that moderate temperatures and low-intensity ultrasonication lead to minor structural damage and enhanced functional properties suitable for food applications.

Regarding the polydispersity of the CSM, a polydispersity index was computed as the ratio of $M_{w}$ and $M_{v}$ values obtained in this work, which resulted in $PDI_{v}=M_{w}/M_{v}=2.52$ indicating a highly polydisperse CSM sample. It is important to note that $M_{w}/M_{v}<M_{w}/M_{n}$ since $M_{n}<M_{v}<M_{w}$. Recently, Li et al. [16] reported a wide molecular weight distribution (*M_w_/M_n_* =3.52) for purified chia seed gum determined by size exclusion chromatography, suggesting that ethanol precipitation, used here as well, increases the proportion of low-molecular-weight components, broadening the molecular distribution compared with crude mucilage (*M_w_/M_n_*=1.15). Since the calculated $PDI_{v}$ indicates a wide molecular-weight distribution, the rheological behavior of CSM dispersions is expected to be dominated by high molecular-weight chains, which have longer relaxation times than those of low molecular weight [35]. Particularly, in the case of microgels, the high-molecular-weight chains are primarily responsible for a strong elastic response, as they are very prone to forming entanglements. Thus, the presence of shorter chains in a polydisperse sample will weaken the three-dimensional network of microgels.

### 2.8. Functional Properties of Optimized-CSM: Water Holding Capacity (WHC) and Oil Holding Capacity (OHC)

The functional properties of optimized-CSM (Table 3) reflect its structure–function relationships, which are controlled by composition and extraction conditions. The Water Holding Capacity (WHC) reached 106.12 ± 0.24 g/g (dry basis) [20,26], consistent with previously reported values for chia mucilage and indicative of a highly hydrated polymer network. For example, Segura-Campos [36] reported 110.5 g/g for partially defatted chia gum, and 108.28 g/g was achieved through microwave-assisted extraction (MAE) [15]. Furthermore, this value exceeds the 95.6 g/g reported by Punia and Dhull [26] using conventional centrifugation. Such behavior is primarily attributed to the high TDF content (75.87%) and the abundance of hydrophilic functional groups (hydroxyl and carboxylate), which promote strong polymer–water interactions.

Structurally, CSM consists of elongated chains and microfibrillar domains that swell upon hydration, forming a three-dimensional network capable of entrapping large amounts of water [37]. These helical conformations act as nanoaggregates with a high density of active sites, enabling optimized-CSM to physically trap “hydrodynamic water” within a dense matrix. The SFAE method likely enhanced the accessibility of hydrophilic sites (hydroxyl and carboxyl groups), allowing the mucilage to retain more than 100 times its weight in water, even under centrifugal stress.

In contrast, the Oil Holding Capacity (OHC) was relatively low (3.13 ± 0.04 g/g), suggesting limited hydrophobic interactions. This result aligns with the predominantly polysaccharidic nature of the system and its moderate protein content. The value is considerably lower than those reported in other studies (22.3 g/g to 34.31 g/g) [11,25,26,27,35,38].

#### Emulsifying Properties of Chia Seed Mucilage

The emulsifying performance of the optimized-CSM, expressed as the emulsion activity index (EAI) and emulsion stability index (ESI), were 62.50 ± 0.12% and 49.32 ± 0.48%, respectively. These values highlight the multifunctional behavior of CSM, which can be attributed to synergistic interactions between residual proteins that can promote interfacial adsorption and reduction in interfacial tension, while polysaccharide chains provide steric stabilization by forming hydrated, viscoelastic layers that limit droplet coalescence and flocculation [39,40,41]. From a structural perspective, this behavior can be interpreted within a microgel framework: hydrated polymer domains act as soft, deformable colloidal particles that absorb at the oil–water interface, deform and interpenetrate, and form viscoelastic interfacial layers. A similar mechanism, known as Pickering-type stabilization, enhances emulsion stability. It should be noted that this interpretation is based on functional parameters and is supported by the literature, as interfacial characterization was not performed in this study.

For instance, Wang et al. [13] reported a maximum ESI of ~58% at a mucilage concentration of 1.0% when extracted at 50 °C. However, increasing the extraction temperature to 80 °C reduced ESI to a range of 38–42% and diminished emulsifying activity, emphasizing the sensitivity of interfacial functionality to processing conditions. Compared with other chia mucilage extracts, the ESI of optimized-CSM is slightly lower than the values reported by Capitani et al. [40] (78.40% with corn oil) and by Coorey et al. [20] (EAI 61.5%, and ESI 69.83%). Such variations are expected and largely attributed to methodological differences, such as oil type and homogenization conditions [39].

Furthermore, the emulsifying performance of optimized-CSM (62.50%) is comparable to that of commercial guar gum (62.33%) and superior to that of gelatin (57%) under similar conditions [20]. These results demonstrate that the controlled SFAE process preserves the molecular features required for both bulk rheological functionality and interfacial activity, reinforcing the potential of chia mucilage as a tunable, structure-driven hydrocolloid for advanced food applications.

### 2.9. Viscoelastic and Viscoplastic Flow Properties of Optimized-CSM

The rheological properties of polysaccharides are intrinsically linked to their molecular weight, conformation, and concentration [42]. In the following section, we describe the viscoelastic and steady-state flow behavior of the CSM dispersions and microgels.

**Figure 9:**
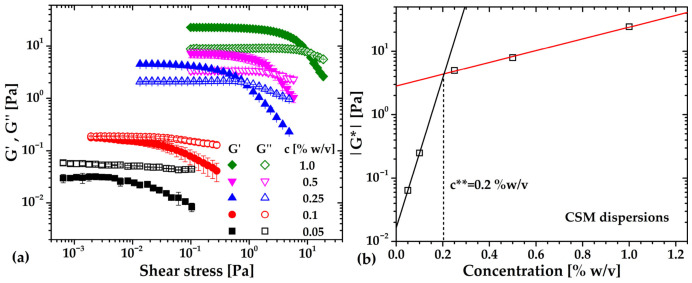
(**a**) Amplitude sweeps and (**b**) complex modulus of viscoelastic and viscoplastic chia seed mucilage (CSM) dispersions as a function of concentration.

Figure 9a depicts the amplitude sweep of CSM across concentrations from 0.05 to 1% (*w*/*v*). At concentrations ≤ 0.1%, the loss modulus (G″) exceeds the storage modulus (G′), indicating viscoelastic fluid behavior. At concentrations ≥ 0.25%, G′ > G″ signifying a transition from viscoelastic to gel-like behavior. In all cases, CSM microgels exhibited weak gel or jammed-state characteristics, with G′/G″ ratios ≥ 3, which is attributed to the wide polydispersity of the optimized-CSM, i.e., to the presence of the low molecular weight chains that give rise to weaker gel networks [35]. These results are consistent with previous reports on CSM powders, regardless of the extraction method or molecular weight [12,43]. As for the fluidization point, or crossover between G′ and G″, increases with concentration, indicating a slowdown in microgel fluidization; the same occurs for G′, which in turn indicates a stronger but still soft structure of the CSM microgels. In general, such behavior is typical of polysaccharide-based microgels used to soften the structure and tailor the rheological responses in blended systems [43,44,45].

To complete this analysis, the jamming concentration (c**) was estimated by plotting the complex modulus (G*) against concentration (Figure 9b). The two linear fits intersected at c** = 0.2% (*w*/*v*), marking the onset of solid-like behavior (a yield-stress fluid) and the formation of a network of densely packed polymer chains.

**Figure 10:**
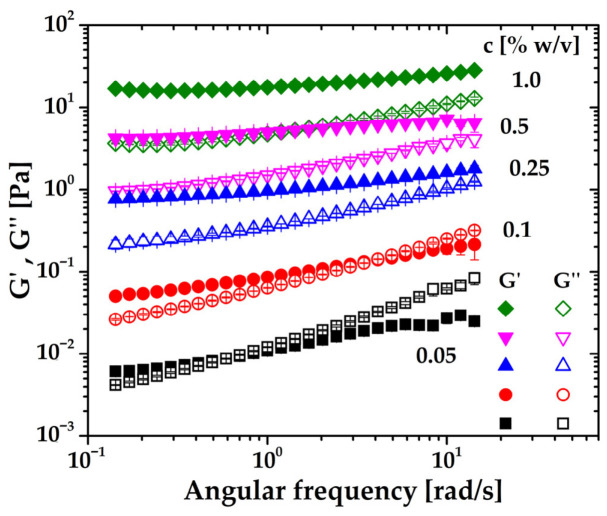
Frequency sweeps of viscoelastic and viscoplastic chia seed mucilage (CSM) dispersions at different concentrations.

The frequency sweeps of the viscoelastic and viscoplastic CSM aqueous dispersions and microgels are shown in Figure 10. At concentrations of *c =* 0.05 and 0.1% (*w*/*v*), G′ and G″ crossed over at ω = 0.6 and 3.5 rad/s, respectively. Below these points, G″ dominated, while above them G′ prevailed, typical of viscoelastic fluids. Interestingly, the anomalous dominance of G′ at lower frequencies has been reported for other CSM extracts [43], suggesting a viscoelastic behavior strongly dominated by the elastic response of the CSM coils. Here, this behavior is attributed to the high polydispersity of the extract (*M_w_*/*M_v_* = 2.52), similar to highly polydisperse polymer melts and wormlike micelles [46], or to a stiff, rod-like conformation of CSM chains [12], which may respond differently from spherical or soft coils in solution.

Regarding the CSM dispersions from *c =* 0.25 to 1% (*w*/*v*), the moduli never cross; however, in these cases, G′ always dominates G″, and again G′/G″ = 3 at much, which indicates the formation of a weak gel network. At higher concentrations (≥0.25% *w*/*v*), G′ consistently dominates G″, confirming weak gel network formation.

**Figure 11:**
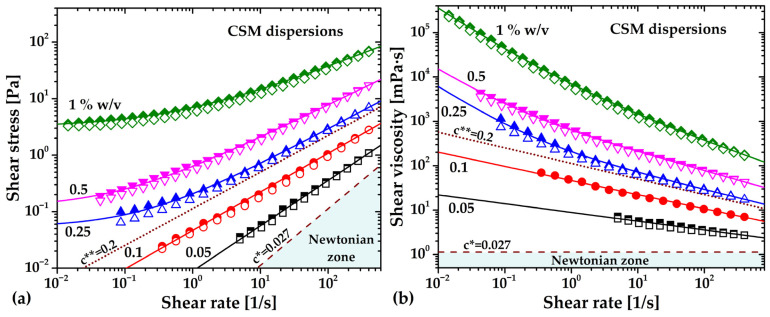
(**a**) Flow and (**b**) viscosity curves of viscoelastic and viscoplastic chia seed mucilage (CSM) dispersions at different concentrations. The dashed line represents the predicted viscosity at c* = 0.027% (*w*/*v*) using the quadratic model in Figure 6b. The dotted line is an estimated power law fluid for c** = 0.2% (*w*/*v*) from the polynomial fittings in Figure 12.

Steady-flow behavior was analyzed using flow and viscosity curves (Figure 11). The curves were constructed for up (filled symbols) and down (hollow symbols) shear-rate cycles. Shear-thinning behavior is observed at relatively low CSM concentrations, i.e., at c = 0.05 and 0.1% (*w*/*v*), which was found to be well-described by the Ostwald de Waele ($\tau =k{\dot{\gamma }}^{n}$ where k and n are the consistency and power-law index, respectively) or power law model (solid lines between c* and c**). As concentration increases above c**, the shear-thinning behavior transitions to a viscoplastic regime, well described by the Herschel–Bulkley model (see solid lines, $\tau =\tau_{y}+k{\dot{\gamma }}^{n}$ where $\tau_{y}$ is the yield stress and k and n are power law parameters), i.e., CSM-concentrated dispersions exhibit a yield stress. The yield stress is the threshold that must be exceeded for the material to undergo non-reversible deformation, that is, to flow; below the yield stress, the material deforms reversibly as an elastic solid. In general, the yield stress increases with concentration, a common response among gel-forming polysaccharides [11]. This change in rheological response is consistent with the amplitude and frequency sweeps shown in Figure 9a and Figure 10. On the other hand, a small decrease in viscosity for all the CSM dispersions is observed in Figure 11b from the up-and-down shear rate ramps, with 10% at much in the studied range. This is indicative of time-dependent or thixotropic behavior; nevertheless, a complete analysis of thixotropy may be the aim of future research. In general, the rheological response, dependent on concentration, is either shear-thinning or viscoplastic and is consistent with that reported for other CSM extracts with different molecular weights and proximate compositions [12,43].

**Figure 12:**
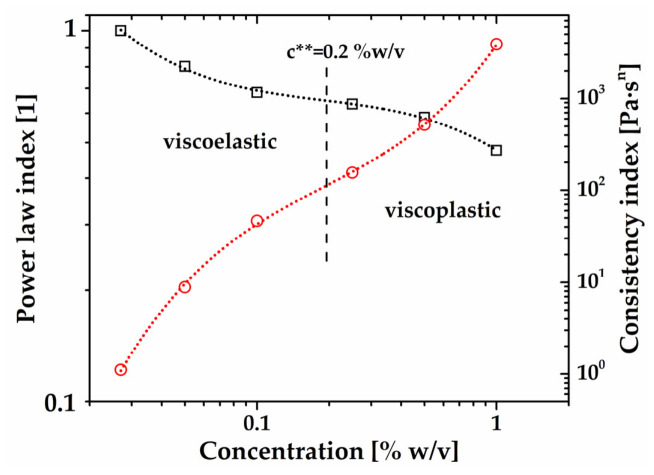
Power law, *n*, and consistency index, k, of the shear thinning and viscoplastic chia seed mucilage (CSM) dispersions as a function of *c*. The first point in the *k* vs. *c* curve corresponds to the predicted dynamic viscosity at c* = 0.027% (*w*/*v*) using the quadratic function in Figure 11b.

Finally, for completeness, the consistency (*k*) and power law (*n*) values of the shear-thinning and viscoplastic CSM dispersions are plotted in Figure 12 as a function of concentration. These material properties were determined from experimental steady-shear flow data fitted with the power law and Herschel–Bulkley models (solid lines in Figure 11). They are used as an alternative method to estimate c**. Briefly, both *n* and *k* follow a cubic polynomial trend. There is an inflection point around c** = 0.195% (*w*/*v*), which agrees with the value of c** = 0.2% (*w*/*v*) obtained by plotting G* against *c*. Note that different concavities may be associated with different rheological responses, and, particularly, the value of *k* starts to diverge above c**, as expected.

## 3. Conclusions

The optimized sonication–freezing-assisted extraction method proved effective in isolating chia seed mucilage (CSM) while preserving structural integrity and well-defined molecular characteristics, as evidenced by consistent intrinsic viscosity values and reliable molecular weight estimates. The system displayed predictable hydrodynamic behavior in dilute regimes, transitioning into soft, solid-like networks with a yield stress above the critical overlap concentration, indicative of microgel-like organization. These rheological properties are inherently linked to the chemical composition of CSM, particularly its high polysaccharide content and the presence of ionized carboxylate groups, which confer a polyelectrolyte nature.

This study establishes a clear structure–function relationship, showing that interactions among extraction conditions, molecular composition, and concentration influence both the rheological behavior and functional performance of CSM. The results highlight chia mucilage as a promising, tunable hydrocolloid for advanced applications, emphasizing the importance of controlled processing strategies to tailor its molecular architecture and functionality to meet specific industrial requirements.

Future research should address the molecular and interfacial mechanisms governing the techno-functional properties of chia seed mucilage. This includes the characterization of zeta potential, particle size distribution, and interfacial rheology, as well as the analysis of molecular mass distribution and the role of uronic acids and total sugars in defining rheological behavior. Furthermore, studies should explore the scalability of the extraction process for industrial applications and evaluate the performance of CSM in complex food systems, pharmaceutical formulations, and biodegradable materials to validate its applicability at larger scales.

## 4. Materials and Methods

### 4.1. Materials

Chia seeds (*Salvia hispanica* L.) were obtained commercially from Grupo Diquitra S.A. de C.V. (Estado de México, Mexico) and stored in a dry, dark environment at room temperature prior to extraction. Commercial chia seed oil (density of 0.87 g/mL) was provided by Jevica (Oleosdey S. de R.L., Tlaxcala, Mexico). The Total Dietary Fiber Assay Kit (DF-100A, Sigma-Aldrich Chemie, Buchs, Switzerland) and all other chemical reagents employed in this study were of analytical grade and purchased from Sigma-Aldrich (St. Louis, MO, USA).

### 4.2. Extraction and Purification of the Chia Seed Mucilage (CSM)

CSM was extracted using a sonication–freezing-assisted method. The effects of hydration temperature, freezing time, and sonication duration on mucilage yield were evaluated through a multifactorial experimental design. The selection of these factors and their ranges was based on their mechanistic considerations related to mass transfer, structural modifications, and polymer stability, as well as bibliographic evidence.

Chia seeds were hydrated in distilled water at a 1:66 (*w*/*v*) ratio and stirred at 700 rpm for 1 h at 40, 60, and 80 °C. These temperatures were selected to ensure adequate ice crystal formation, thereby promoting structural rupture of the seed coat. The resulting dispersions were subsequently sonicated in an ultrasonic bath (Branson 2800; CPX-952-216R, Branson Ultrasonic Corp, Danbury, CT, USA, operating at 40 kHz and 110 W). Sonication times of 30, 45, and 60 min were chosen to represent a moderate-energy-input region, where cavitation enhances mass transfer without inducing excessive polymer degradation.

To stabilize the biopolymer and facilitate mechanical detachment of mucilage from the seed coat via ice crystal formation, samples were immediately frozen at −20 °C for 0, 24, and 48 h. After defrosting, the hydrated dispersions were passed through a hand-operated rotary sifter (Mainstays, Dongguan, China) with an approximately 0.8 mm stainless-steel mesh to remove seeds and maximize mucilage recovery. A subsequent filtration step was performed using a standardized 0.4 mm mesh sieve to eliminate residual fine particulate matter.

The filtrate was transferred to a separating funnel, where an ethanol–acetone solution (70:30, *v*/*v*) was added at a solvent:sample ratio of 2:1 to precipitate the insoluble polysaccharide fraction. The resulting precipitate was collected and dried in a convection oven at 40 °C for 12 h. The dried mucilage was ground using a commercial blade grinder (F2034251, KRUPS, Solingen, Germany), sieved through a 0.4 mm mesh to ensure a uniform particle size, and stored in hermetically sealed glass jars at room temperature until further analysis.

#### 4.2.1. Extraction Yield

The extraction yield was calculated as a percentage according to Equation (2):

(2) $$Y\left(\%\right)=\frac{W_{2}}{W_{1}}\times 100$$

where Y (%) is the percent yield, *W*_2_ is the weight of the dried mucilage extract, and *W*_1_ is the initial weight of the chia seeds.

#### 4.2.2. Experimental Design and Statistical Analysis

The extraction process for chia seed mucilage (CSM) was optimized using a Box–Behnken Design (BBD) combined with Response Surface Methodology (RSM) to evaluate the effects of independent variables (sonication time, hydration temperature, and freezing time) on the extraction yield, including linear, quadratic, and interaction effects among these factors.

A three-factor, three-level BBD was used, comprising 17 experimental runs (Table A1), including five replicates at the central point to estimate experimental error and ensure process reproducibility. Experimental data were processed and analyzed using Design-Expert^®^ software version 10 (Stat-Ease Inc., Minneapolis, MN, USA). The statistical significance of the model and its terms were evaluated using Analysis of Variance (ANOVA) at a 95% confidence level (*p* < 0.05).

#### 4.2.3. Yield Extraction Optimization

Optimization was conducted in Desing-Expert^®^ using the Response Optimizer tool to identify optimal processing conditions. Model validity was assessed by replicating the optimal conditions and comparing yields with the predicted values.

### 4.3. Physicochemical Properties of CSM Obtained Under Optimized Conditions

The mucilage obtained under optimized extraction conditions was subjected to comprehensive physicochemical characterization using standardized methods to establish relationships between process variables and mucilage quality.

#### 4.3.1. Colorimetric Parameters

Colorimetric properties were measured using a digital colorimeter (model LS173, Shenzhen Linshang Technology Co., Ltd., Shenzhen, China) equipped with a full-spectrum LED light source and operated under diffuse illumination geometry (D/8°), with the specular component included (SCI). Before measurements, the instrument was verified using the manufacturer’s calibration plate and certified reference standards. The calibration certificate provided by the manufacturer specifies reference values for red $\left(Y_{0}=12.92,x_{0}=0.5029,y_{0}=0.3321\right)$, green $\left(Y_{0}=44.21,x_{0}=0.2708,\;y_{0}=0.3360\right)$, yellow $\left(Y_{0}=48.28,x_{0}=0.4405,y_{0}=0.4456\right)$, blue $\left(Y_{0}=28.48,x_{0}=0.2540,y_{0}=0.2918\right)$, and white $\left(Y_{0}=84.91,x_{0}=0.3133,y_{0}=0.3313\right)$. In this context, $x_{0}$ and $y_{0}$ represent the chromaticity coordinates of the standard reference white, while $Y_{0}$ denotes its luminance (lightness). These calibration procedures ensured the accuracy and reproducibility of the colorimetric measurements.

Mucilage samples (3 g) were placed in sterile plastic Petri dishes (60 × 15 mm), and the surfaces were gently leveled to ensure a uniform measurement area. Color parameters were recorded in the CIE-Lab* color space, including lightness (L*), and chromatic coordinates (±a* and ±b*).

#### 4.3.2. Proximate Chemical Analysis of Optimized-CSM

The proximate composition of optimized-CSM was determined following standard methods established by the American Oil Chemists Society (AOCS) and the Association of Official Analytical Chemists (AOAC). Moisture content was determined using the AOCS Ba 2a-38 method, total dietary fiber was quantified using the AOAC method 985.29, and ash content was determined by the AOAC method 923.03. Total nitrogen content was analyzed by the Kjeldahl method, and protein content was calculated using a nitrogen conversion factor of 6.25. Carbohydrate content was estimated as nitrogen-free extract (NFE) by difference, according to AOAC guidelines, using Equation (3):

(3) $$NFE\;\left(\%\right)=100-\left(\mathrm{lipids}+\mathrm{protein}+\mathrm{moisture}+\mathrm{total}\;\mathrm{dietary}\;\mathrm{fiber}+\mathrm{ash}\right)$$

In Equation (3), the values for each proximate composition parameter correspond to their respective percentages.

#### 4.3.3. Fourier Transform Infrared Spectroscopy

CSM samples were characterized using a Frontier FTIR spectrometer (PerkinElmer, Waltham, MA, USA) equipped with an Attenuated Total Reflectance (ATR) accessory. Spectra were recorded at room temperature over the wavenumber range of 4000–600 cm^−1^, with a resolution of 4 cm^−1^ and 60 scans per measurement. Background correction was performed using air before each measurement.

### 4.4. Preparation of CSM Solutions for Intrinsic Viscosity, Refractive Index, and SLS Measurements

A stock aqueous solution of CSM was prepared at a concentration of 0.050 g/dL. Precisely 0.050 ± 0.001 g of the CSM powder was weighed using an analytical balance (OHAUS, Parsippany, NJ, USA) and dispersed in 30 mL of reagent-grade deionized water (Meyer). The dispersion was magnetically stirred at 25 °C (Cole–Palmer, Vernon Hills, IL, USA) at approximately 600 rpm for 12 h to ensure homogeneity. After that, insoluble solids remained suspended, potentially leading to an overestimation of viscosity. To remove these residues, the 30 mL dispersion was centrifuged at 1000 rpm for 30 s. The supernatant was recovered and diluted to a final volume of 100 mL in a volumetric flask. The centrifuge tubes with solids were dried at 60 °C for 24 h in an oven (ECOSHELL, Santiago, Chile). Tubes were weighed before and after drying to determine the mass of insoluble residue. This insoluble mass was subtracted from the initial CSM powder weight difference, which was used to calculate the final concentration of the CSM stock, which was 0.039 g/dL. From this stock, five dilutions (25 mL each) were prepared at concentrations of 0.0024, 0.0032, 0.0040, 0.0048, and 0.0056 g/dL using micropipettes (1 mL and 100 μL capacities).

#### 4.4.1. Double-Gap Viscometry of CSM Solutions

Intrinsic viscosity and viscosity-average molecular weight were measured using a controlled stress–strain rotational rheometer (MCR 302, Anton Paar, Graz, Austria) equipped with a double-gap concentric cylinder (Couette) geometry, specifically designed for accurate characterization of very low-viscosity fluids. The methodology followed that of Marín-Santibáñez et al. [7] for chitosan and xanthan gum solutions. Briefly, viscosity curves were measured over a shear rate ranging from 100 to 400 s^−1^ in triplicate at 25 °C for the five dilutions described above. The resulting viscosity data were used to compute the *M_v_* value via the Mark–Houwink–Sakurada equation (Appendix A).

#### 4.4.2. Refractive Index and Static Light-Scattering Measurements

Determination of the weight-average molecular weight, *M_w_*, requires prior measurement of the refractive index of dilute polymer solutions. The refractive index, *n*, of the five dilutions (Section 4.4), all exhibiting Newtonian flow behavior, was determined using an Abbe refractometer (Abbemat 550, Anton Paar, Graz, Austria) to compute the derivative *dn/dc*. For each measurement, 0.3 mL of solution was placed in the refractometer with a micropipette. The value of *dn/dc* was used to obtain *Kc/R_θ_* values for Debye plot construction and *M_w_* determination (Appendix B) throughout SLS measurements. The refractometer can measure refractive index increments as small as 1 × 10^−6^, making it ideal for extremely dilute polymer solutions (a few milligrams per 100 dL) and for accurate determination of *M_w_*.

Static light-scattering (SLS) measurements were conducted using a Litesizer 701 instrument (Anton Paar, Graz, Austria). Clean quartz cuvettes were filled with 1.2 mL and analyzed at 25 °C.

### 4.5. Functional Properties of Optimized-CSM

#### 4.5.1. Water Holding Capacity (WHC) and Oil Holding Capacity (OHC)

WHC was determined following the method of Wang et al. with slight modifications. Briefly, 0.5 g of CSM was mixed with 25 mL of distilled water. OHC was determined using a commercial chia oil. Samples were homogenized with a vortex mixer (Vortex-Genie 2, Scientific Industries, Bohemia, NY, USA) at 2420 rpm for 1 min, then incubated at 4 °C for 24 h to ensure complete absorption. The mixtures were centrifuged at 2000× *g* for 10 min using a benchtop centrifuge (L420, Labtron Equipment Ltd., Surrey, UK). The supernatant was discarded, and the precipitate was collected. WHC and OHC were calculated using Equations (4) and (5):

(4) $$WHC\;\left(\frac{g}{g}\right)=\frac{\mathrm{Sample}\;\mathrm{weight}\;\mathrm{after}\;\mathrm{water}\;\mathrm{absorption}\;-\;\mathrm{Sample}\;\mathrm{weight}\;\mathrm{before}\;\mathrm{water}\;\mathrm{absorption}}{\mathrm{Sample}\;\mathrm{weight}\;\mathrm{before}\;\mathrm{water}\;\mathrm{absorption}}$$

(5) $$OHC\left(\frac{g}{g}\right)=\frac{\mathrm{Sample}\;\mathrm{weight}\;\mathrm{after}\;\mathrm{oil}\;\mathrm{absorption}-\mathrm{Sample}\;\mathrm{weight}\;\mathrm{before}\;\mathrm{oil}\;\mathrm{absorption}}{\mathrm{Sample}\;\mathrm{weight}\;\mathrm{before}\;\mathrm{oil}\;\mathrm{absorption}}$$

#### 4.5.2. Emulsifying Capacity (EC) and Emulsifying Stability Index (ESI)

Emulsifying properties of CSM were evaluated according to Coorey et al. [20] with minor modifications. For this EC determination, 0.5 g of CSM was dispersed in 50 mL of distilled water at 60 °C under magnetic stirring at 700 rpm for 30 min. The dispersion was homogenized using an Ultra-Turrax (IKA T18 basic, IKA^®^-Werke GmbH & Co. KG, Staufen im Breisgau, Germany) at 24,000 rpm for 2 min at room temperature. An oil-in-water emulsion was prepared by adding 50 mL of commercial chia oil to the hydrated mucilage solution and homogenizing for 10 min at 15,500 rpm. The emulsion was centrifuged at 1410× *g* for 15 min, and the volume of the emulsified layer was recorded.

Emulsion stability was assessed by heating the emulsion at 80 °C for 30 min, cooling to room temperature, and centrifuging at 1410× *g* for 15 min. ESI was expressed as the percentage of the remaining emulsified layer relative to the initial emulsion volume (50 mL). EC and ESI were calculated using the following equations:

(6) $$EC\left(\%\right)=\frac{\mathrm{Final}\;\mathrm{volume}\;\mathrm{of}\;\mathrm{the}\;\mathrm{emulsified}\;\mathrm{layer}}{\mathrm{Initial}\;\mathrm{total}\;\mathrm{emulsion}\;\mathrm{volume}}\times 100$$

(7) $$ESI\;\left(\%\right)=\frac{\mathrm{Final}\;\mathrm{volume}\;\mathrm{of}\;\mathrm{the}\;\mathrm{heated}\;\mathrm{emulsified}\;\mathrm{layer}}{\mathrm{Initial}\;\mathrm{heated}\;\mathrm{emulsion}\;\mathrm{volume}}\times 100$$

### 4.6. Rheometry of CSM Dispersions and Microgels

Rheometric characterization of optimized-CSM dispersions and hydrogels from 0.05 to 1% (*w*/*v*) was conducted using a stress-controlled rotational rheometer (MCR 302). For low viscosity samples, two concentric cylinder (Couette) geometries were attached to the MCR 302: a DIN standard Couette, and a double-gap Couette geometry for c < 0.2% (*w*/*v*). A parallel-plate geometry with sandpaper was used to avoid the effect of wall slip on the rheometrical measurements of CSM hydrogels for c > 0.2% (*w*/*v*). The rheometrical experiments consisted of ramps at controlled shear rates ranging from 0.001 to 400 1/s to build flow and viscosity curves for CSM dispersions and hydrogels. On the other hand, amplitude sweeps were performed while controlling shear stress from 0.0005 to 20 Pa at a constant frequency of 1 Hz. Once the linear viscoelastic region (LVR) for each CSM dispersion and hydrogel was determined, a frequency sweep was performed at a constant stress within the LVR, varying the angular frequency from 0.1 to 20 rad/s. All experiments were performed in triplicate and at a constant temperature of 25 ± 0.2 °C. The temperature was controlled by a Peltier-type heating system.

## Figures and Tables

**Table 1:** Summary of reported extraction methodologies, yields, and molecular characterization of chia seed mucilage (CSM) from the literature.

Extraction and Purification	Yield [%]	Molecular Weight [g/mol]	Method	MHS Parameters K [mL/g]	Reference
Hydration (1:20) + centrifugation + double ethanol precipitation (1:3 *v*/*v*, 99%) + Freeze-drying (Lyophilization)	1.02	*M_w_* = 4.9 ± 0.2 × 10^5^ PDI = 1.02	SEC-MALLS-Viscometry	K = 1.95 α = 0.45	[11]
Hydration + Lyophilization Soxhlet + Precipitation with boiling 80% ethanol + centrifugation + Acetone precipitation + Dialysis + Lyophilization	3.80	*M_w_* = 2.34 × 10^6^ *M_n_* = 1.07 × 10^6^ PDI = 2.2	SEC with RI detector, PEG/PEO as standards	[η] = 16.63 dL/g K = 1.52 × 10^−4^ α = 0.803 k_H_ = 0.736	[12]
Hydration (1:20) + Boiling water (5 min) + Stirring (1300 rpm) + High-speed shearing (3000 rpm, 2 min) + Centrifugation + Ethanol precipitation + vacuum filtration + Lyophilization	6.25	CCSM *M_w_* = 3.99 × 10^5^ *M_n_* = 3.46 × 10^5^ PDI = 1.151 PCSM *M_w_* = 1.72 × 10^5^ *M_n_* = 4.88 × 10^4^ PDI = 3.519	SEC with online viscometer (universal calibration)	N. R.	[16]

*M_w_*: Weight-average molecular weight; *M_n_*: Number-average molecular weight; PDI: Polydispersity index; SEC: Size Exclusion Chromatography; RI: Refractive index; PEG/PEO: Polyethylene glycol/oxide; MALLS: Multi-Angle Laser Light Scattering; MHS: Mark–Houwink–Sakurada constants; K: Empirical constant; α: Function of polymer geometry (shape factor); k_H_: Huggins coefficient; N. R.: Not reported, CCSM: Crude Chia Seed Mucilage, PCSM: Purified Chia Seed Mucilage.

**Table 2:** Proximate composition of chia seed mucilage (CSM) and extraction yield (dry basis).

Yield (%)	Moisture (%)	Ash (%)	Lipids (%)	Protein (%)	NFE (%)	TDF (%)	Ref
3.39 ± 0.24	12.23 ± 0.16	9.08 ± 0.01	2.95 ± 0.02	8.71 ± 0.02	3.39 ± 0.81	75.87 ± 0.76	Optimized-CSM
3.80	3.90 ± 0.30	0.80 ± 0.10	0.60 ± 0.10	2.60 ± 0.20	93.80 ± 0.50	N. R	[16]
2.11 ± 0.07	7.03 ± 0.08	7.68 ± 0.06	1.22 ± 0.09	0.73 ± 0.08	90.38 ± 0.13	N. R	[11]
1.20 ± 0.05	N. R	N. R	N. R	3.80 ± 0.20	95.0 ± 1.5	N. R	[15]
6.25	5.52 ± 0.14	7.86 ± 0.38	N. D	14.44 ± 0.14	51.85 ± 1.26	N. R	[14]
3.68	N. R	2.80 ± 0.23	5.17 ± 5.34	25.2 ± 0.77	66.83 ± 5.40	N. R	[12]

N. R: No reported, N. D: Not detected, NFE: Nitrogen-free extract, TDF: Total dietary fiber. All compositional values are expressed on a dry basis (D.B) except for moisture.

**Table 3:** Techno-functional properties of optimized-CSM.

Functional Property	Values
Water Holding Capacity (WHC, g/g)	106.12 ± 0.24
Oil Holding Capacity (OHC, g/g)	3.13 ± 0.04
Emulsion activity index (EAI, %)	62.50 ± 0.12
Emulsion stability index (ESI, %)	49.32 ± 0.48

Values are expressed as mean ± standard deviation (n = 3).

## Data Availability

The data presented in this study are available on request.

## Abbreviations

The following abbreviations are used in this manuscript:

SFAE	Sonication–Freezing-Assisted Extraction
CSM	Chia Seed Mucilage
SLS	Static Light Scattering
FTIR	Fourier transform infrared
EAI	Emulsion activity index
ESI	Emulsion stability index
PDI	Polydispersity index
*M_w_*	Weight-average molecular weight
*M_n_*	Number-average molecular weight
*M_v_*	Viscosity-average molecular weight
SEC	Size Exclusion Chromatography
MALLS	Multi-Angle Laser Light Scattering
NIST	National Institute of Standards and Technology
TDF	Total dietary fiber
WHC	Water Holding Capacity
OHC	Oil Holding Capacity
BBD	Box–Behnken Design
ANOVA	Analysis of Variance
SCI	Specular Component Included
AOCS	American Oil Chemists Society
AOAC	American Official Analytical Chemists
NFE	Nitrogen-free extract
ATR	Attenuated Total Reflectance
EC	Emulsifying capacity
LVR	Linear viscoelastic region

## Appendix A

### Appendix A.1. Basis of Intrinsic Viscosity

To determine the viscosity-average molecular weight, *M_v_*, of polymers and polysaccharides, the intrinsic viscosity, [*η*], must first be measured. This requires measuring the dynamic (shear) viscosity of a series of polymer solutions exhibiting Newtonian flow behavior and constructing the Huggins [47] and Kraemer [48] curves, defined by the following equations:

(A1) $$\frac{\eta_{sp}}{c}=\left[\eta \right]+k_{H}{\left[\eta \right]}^{2}c$$

(A2) $$\frac{\eta_{sp}}{c}=\left[\eta \right]+k_{H}{\left[\eta \right]}^{2}c$$

where *c* is the polymer concentration, $\frac{\eta_{sp}}{c}$ is the reduced specific viscosity, and $\frac{{ln(\eta }_{rel})}{c}$ is the inherent viscosity. In Equations (A1) and (A2), the relative viscosity, $\eta_{rel}$, and the specific viscosity, $\eta_{sp}$, are calculated from the polymer solution viscosity, η, and the solvent viscosity, $\eta_{0}$, as follows:

(A3) $$\eta_{rel}=\;\frac{\eta }{\eta_{0}}$$

(A4) $$\eta_{sp}=\eta_{rel}-1$$

where [η] denotes the intrinsic viscosity, while *η* represents the dynamic viscosity of the polymer solution. For reliable measurements, both reduced specific viscosity and inherent viscosity must vary linearly with concentration, with both extrapolating to $\left[\eta \right]$ at the origin. This ensures that experimental measurements were free of problems associated with molecular aggregation, ionic effects, or other issues. For many polymers in good solvents, the addition of the Huggins and Kraemer parameters yields $k_{H}+k_{K}\approx 0.5$ [49].

Once $\left[\eta \right]$ is determined, *M_v_* can be calculated using the empirical Mark–Houwink–Sakurada relationship:

(A5) $$\left[\eta \right]=KM_{v}^{\alpha }$$

where *K* and *α* are constants specific to the polymer–solvent system at a given temperature. Particularly, the values of *K* and *α* are obtained from double-logarithmic plots of absolute molecular weight vs. intrinsic viscosity [49].

### Appendix A.2. Double-Gap Couette Viscosimeter

The double-gap Couette geometry consists of a hollow measuring cylinder with radii R_2_ and R_3_, and a special cup with radii R_1_ and R_4_. The cylinder rotates at angular velocity, Ω, under applied torque, M, while the cup remains stationary. Then, the sample is subjected to shear flow. For incompressible fluids under isothermal, steady, and laminar flow, free of chemical reactions, the shear stress, τ, and shear rate, $\dot{\gamma }$, of the fluid in between the gaps of the geometry are determined by using the following equations [50]:

(A6) $$\tau =\frac{M}{4\pi L}\left[\frac{{R_{1}}^{2}+{R_{2}}^{2}}{{R_{2}}^{2}\left({R_{1}}^{2}+{R_{3}}^{2}\right)}\right]$$

(A7) $$\dot{\gamma }=\Omega \left[\frac{{R_{1}}^{2}+{R_{2}}^{2}}{{R_{2}}^{2}-{R_{1}}^{2}}\right]$$

The dynamic viscosity, η, is given by the quotient of Equations (A6) and (A8):

(A8) $$\eta =\frac{m}{4\pi \Omega L}\left[\frac{{R_{2}}^{2}-{R_{1}}^{2}}{{R_{2}}^{2}\left({R_{1}}^{2}+{R_{3}}^{2}\right)}\right]$$

For the double-gap Couette geometry employed in this work, designed according to the standard DIN 54453:1982-01 [51], the following radius ratios are true [52]:

(A9) $$\frac{R_{4}}{R_{3}}=\frac{R_{2}}{R_{1}}\leq 1.15$$

## Appendix B. Basis of Static Light-Scattering Measurements

The weight-average molecular weight (*M_w_*) of polymers can be determined from the intercept of the Debye equation [53]:

(A10) $$\frac{Kc}{R_{\theta }}=\frac{1}{M_{w}}+2A_{2}c$$

where *K* is the optical constant, *R_θ_* is the Rayleigh ratio, and *A*_2_ is the second virial coefficient whose positive or negative sign indicates favorable polymer–solvent or polymer–polymer interactions, respectively. Before applying Equation (A10), the values of *K* and *R_θ_* must be determined from the equations below:

(A11) $$K=\frac{2\pi^{2}}{\lambda^{4}N_{A}}{\left(n_{0}\frac{dn}{dc}\right)}^{2}$$

(A12) $$R_{\theta }=\frac{(I_{s}-I_{0})n_{0}^{2}}{I_{T}n_{T}^{2}}R_{T}$$

where *λ* = 658 nm is the wavelength of the incident light, *N_A_* is the number of Avogadro, *n_0_* stands for the refractive index of the solvent (deionized water in this work), and the derivative *dn/dc* is determined from the linear regime of the curve *n* vs. *c*. On the other hand, *I*_0_ and *I_S_* in Equation (A12) represent the intensities of the light scattered by the solvent and polymer solutions, respectively. *I_T_*, *n_T_*, and *R_T_* = 1.14574 × 10^−5^ cm^−1^ are the dispersed light intensity, refractive index, and Rayleigh ratio of toluene, the reference fluid for light-scattering measurements used in this work.

## Appendix C. Box–Behnken Experimental Design Matrix

**Table A1:** Box–Behnken experimental design matrix in coded levels for the sonication–freezing-assisted extraction (SFAE) of chia seed mucilage.

Experiment	A: Sonication Time	B: Hydration Temperature	C: Freezing Time
1	1	0	1
2	0	1	−1
3	1	−1	0
4	−1	1	0
5	1	0	−1
6	1	1	0
7	−1	0	−1
8	−1	−1	0
9	−1	0	1
10	0	1	1
11	0	−1	−1
12	0	−1	1
13	0	0	0
14	0	0	0
15	0	0	0
16	0	0	0
17	0	0	0

Coded levels represent the actual experimental values for each factor: Factor A (sonication time: −1 = 30 min, 0 = 45 min, 1 = 60 min); Factor B (hydration temperature: −1 = 40 °C, 0 = 60 °C, 1 = 80 °C); Factor C (freezing time: −1 = 0 days, 0 = 1 day, 1 = 2 days).
